# Relationship between premature ejaculation and depression: A PRISMA-compliant systematic review and meta-analysis

**DOI:** 10.1097/MD.0000000000004620

**Published:** 2016-09-02

**Authors:** Yue Xia, Juanjuan Li, Guang Shan, Huijun Qian, Tao Wang, Wei Wu, Jun Chen, Luhao Liu

**Affiliations:** a Department of Urology, Renmin Hospital of Wuhan University, Wuhan, Hubei; b Department of Breast and Thyroid Surgery, Renmin Hospital of Wuhan University, Wuhan, Hubei; c Department of Urology, Longjiang Hospital of Shunde District in Foshan City; d Department of Physiology of Southern Medical University, Guangzhou, Guangdong; e Department of Infertility and Sexual Medicine, the Third Affiliated Hospital of Sun Yat-sen University, Guangzhou; f Department of Urology & Andrology, Minimally Invasive Surgery Center, Guangdong Key Laboratory of Urology, The First Affiliated Hospital of Guangzhou Medical University, Guangzhou, Guangdong, China.

**Keywords:** depression, meta-analysis, premature ejaculation

## Abstract

**Background:**

Premature ejaculation (PE) is the most prevalent male sexual dysfunction. Epidemiologic findings are inconsistent concerning the risk for depression associated with PE.

**Objective:**

The aim of this study was to investigate the potential association between between depression and risk of PE.

**Data sources:**

We conducted a literature search of PubMed, Embase, and the Cochrane Library from these databases’ inception through June 2014 for observational epidemiological studies examining the association between depression on risk of PE.

Study eligibility criteria: Studies were selected if they reported the risk estimates for PE associated with depression.

**Participants:**

patients>18 years of age suffering from PE.

**Interventions:**

a history of depressive disorder.

**Study appraisal and synthesis methods:**

These odds ratios (ORs) were pooled using a random or fixed effects model and were tested for heterogeneity. Subgroup analysis was employed to explore heterogeneity.

**Results:**

Eight trials involving 18,035 patients were included in the meta-analysis. Depression were statistically significantly associated with the risk of PE (OR = 1.63, 95% CI:1.42–1.87). There was no evidence of between-study heterogeneity (*P* = 0.623, *I*^2^ = 0.0%). The association was similar when stratified by mean age, geographical area, study design, sample size, publication year, and controlling key confounders.

**Limitations:**

The severity of depression and PE could not be identified due to unavailable data of trials. No evidence of publication bias was observed.

**Conclusions:**

These findings provide evidence that depression is associated with a significantly increased risk of PE. In addition, more prospective studies are necessary to evaluate the association and identify the ideal treatment.

**Systematic review registration number:**

CRD42016041272

## Abstract 2

Supplemental Digital Content is available in the text

## 1 Introduction

Premature ejaculation (PE) is a common male sexual dysfunction that may adversely affect 20% to 30% of the male population.^[1,2]^ The prevalence of PE did not vary significantly in young and middle-aged men, indicating that no particular age group has consistently been shown to be at greater risk for PE.^[3,4]^ Traditionally, PE can be classified as lifelong or acquired PE. Besides lifelong PE and acquired PE, 2 more types of PE have been proposed: natural variable PE and premature-like ejaculatory dysfunction.^[5–7]^ Men with PE complain about decreased sexual self-confidence and psychological comorbidities. Thus, it seriously impair male health and couples’ sexual relationships.^[8]^

Depression is a common disorder and affects about 26% of women and 18% of men in the United States.^[9]^ Individual psychological factors such as depression, stress, anxiety, and negative cognitive processing are strongly associated with the onset and maintenance of male sexual difficulties. Several studies have shown that impaired sexual function in men with PE is significantly associated with depression.

Gao et al^[10]^ investigated the prevalence of and factors associated with the complaint of PE in China and showed that men with PE were more likely to self-report other sexual dysfunctions (low libido and erectile dysfunction) and psychological disturbances (depression and anxiety) than men without PE. In a study on the epidemiology of depression in men with PE, an association was observed between PE and depression, with an odds ratio (OR) of 1.39.^[11]^

Despite the large number of studies exploring the association between depression and PE, results from previous studies have been inconsistent. The aims of this systematic review and meta-analysis were to quantitatively assess all qualified observational studies that have examined the effect of depression on the risk of PE and to gather more accurate and precise information about this effect.

## 2 Materials and methods

This meta-analysis was conducted according to the Meta-analysis of Observational Studies in Epidemiology (MOOSE) guidelines and the Preferred Reporting Items for Systematic Reviews and Meta-Analyses (PRISMA).^[12,13]^ The protocol for the review is available on PROSPERO (CRD42016041272; http://www.crd.york.ac.uk/PROSPERO). Ethics approval was not needed as this is a secondary literature-based study.

### 2.1 Search strategy

We searched the following databases up to and including June 2014: MEDLINE by PubMed, Embase, Cochrane Central Register of Controlled Trials (Cochrane Library). The following keywords, considering all possible combinations, were used: sexual dysfunction, premature ejaculation, depression, depress, and depressive disorder (for search terms see Supplementary Box 1). Searches were restricted to human studies and articles published in English. All of the references in the relevant articles were screened for any further articles that were not identified in the initial search. All retrieval literatures were independently performed by YX and JJL.

### 2.2 Study selection

We defined the study eligibilities by selecting the patient population, intervention/exposure, comparator, outcome, and study design (PICOS).^[14]^ The PICOS evidence base used consisted of the following combinations: (1) participants: patients >18 years of age suffering from PE. (2) Interventions: a history of depressive disorder. (3) Comparisons: compared with the general population. (4) Outcomes: the diagnosis of PE and measurement of intravaginal ejaculatory latency time (IELT). (5) Study design: any type of observational, cohort or cross-sectional study, and case series. Exclusion criteria were the following: (1) articles not in English; (2) incomplete data availability; (3) review or meta-analysis articles; (4) duplicated or updated data; (5) comments, editorials, letters, and congress reports; animal studies and case reports. In the case of multiple publications based on the same study sample, the most recent publication was included in the analysis. Agreement between investigators was assessed with the Kappa statistic. Disagreement regarding eligibility was resolved by consensus.

### 2.3 Data extraction

All potentially relevant articles were independently evaluated by 2 investigators (YX and JJL), and disagreements were resolved by consensus or consultation with a third author (HQ). Using a standardized form, we recorded procedural characteristics of each study, including the first author's last name, the year of publication, type of design, country of study, number of participants, mean age or age range, the diagnosis criteria for depression, the ascertainment of PE, the case and the control sample sizes, and variables adjusted in the analysis.

### 2.4 Statistical analysis

The odds ratios (ORs) and the corresponding 95% confidence interval (CI) were used as the common measurement for the association between depression and the risk of PE across the studies. The Cochrane's *Q* statistic test and *I*^2^ statistic for heterogeneity were conducted.^[15]^*I*^2^ values of <50% were defined as acceptable; those >50% indicated high levels of heterogeneity. Random-effects models were used in the case of heterogeneity for the meta-analysis; otherwise, fixed-effects models were applied. Sensitivity analyses were conducted to identify studies that excessively contributed to heterogeneity with the leave-one-out method. Publication bias was assessed using Begg's rank correlation test.^[16]^ All analyses were performed using STATA version 12.0 (Stata Corporation, College Station, TX). For all statistical analyses, a 2-sided *P* value of <0.05 was considered statistically significant.

## 3 Results

### 3.1 Literature search

**Figure 1:**
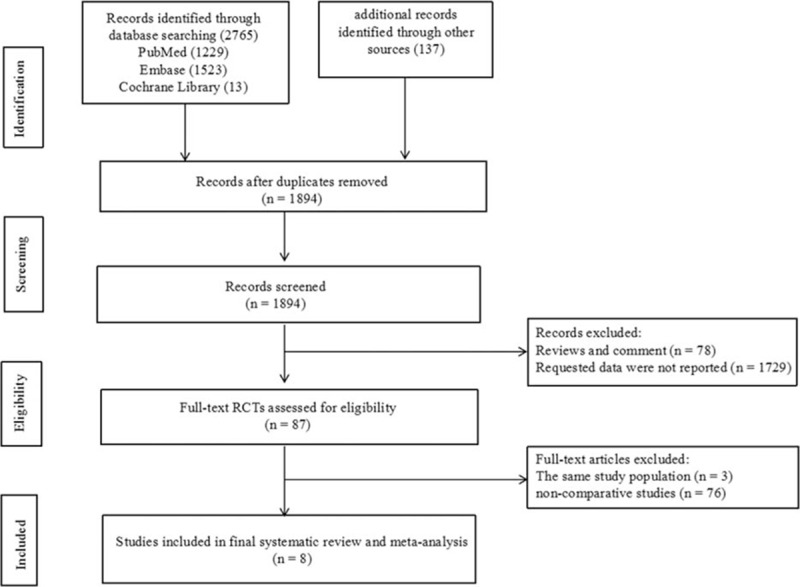
Flowchart of study selection.

The study flow diagram is shown in Fig. 1. After eliminating duplicate publications, we identified 2765 potential articles. After initial screening based on titles and abstracts, 87 publications remained for detailed full-text evaluation. In total, 79 articles were excluded because they did not meet the selection criteria. Finally, 8 published articles^[4,11,17–22]^ were ultimately identified as relevant to our research. Agreement between the reviewers was good (Kappa statistic = 0.68).

### 3.2 Study characteristics

Table 1 depicts the study characteristics and methodology for the 8 studies included in the systematic review. Among these, 2 studies^[11,22]^ were cohort studies, and 6 studies ^[4,17–21]^ were cross-sectional studies. The 9 selected studies contained a total 18,035 subjects. Sample sizes ranged from 270 to 12,133. All studies were published from 2007 to 2014. Three of the included studies^[4,19,22]^ were conducted in the Europe (Germany and Switzerland); 4 were in Asia (Korea, India, and Malaysia)^[11,17,18,20]^, and 1 study was from Australia^[21]^.

**Table 1:**
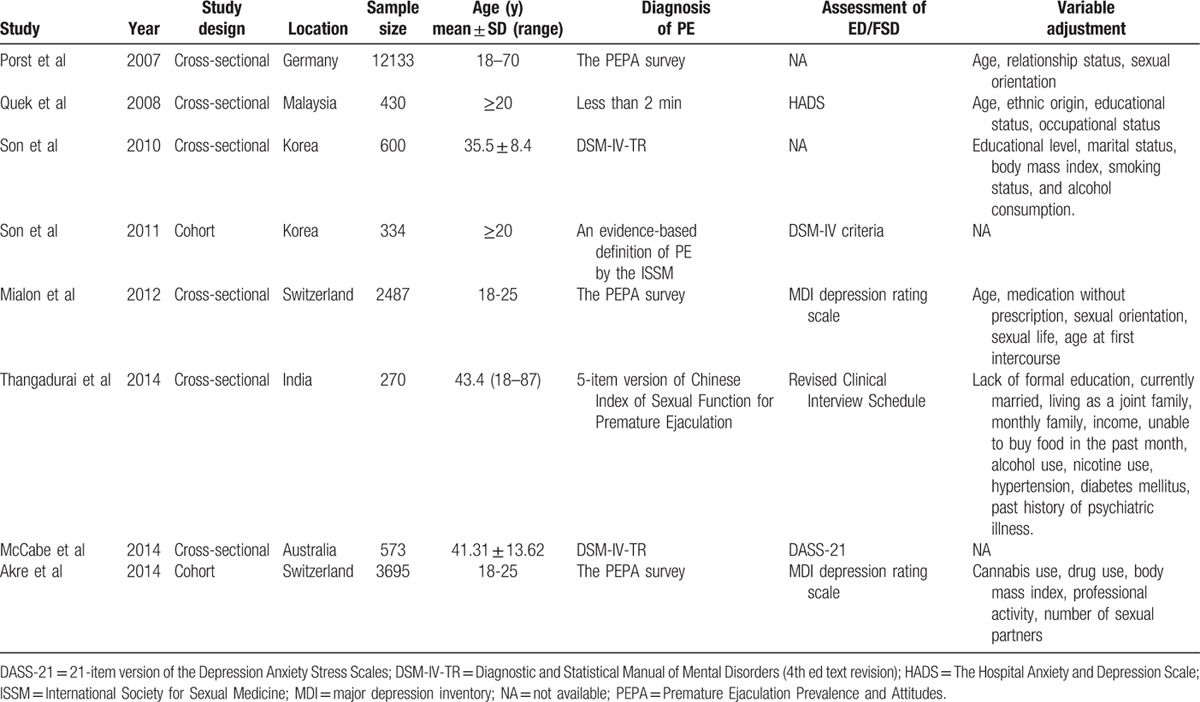
Characteristics of the included studies.

### 3.3 Synthesis of results

**Figure 2:**
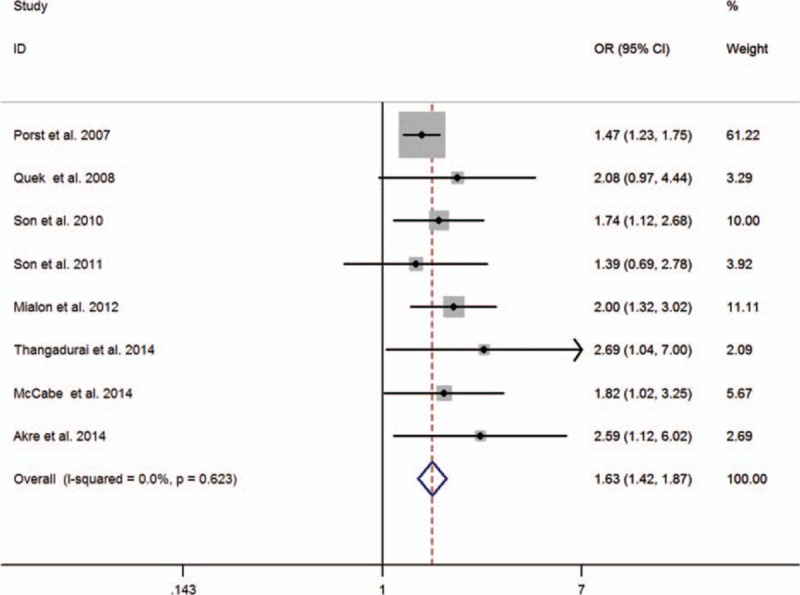
Forest plots of meta-analysis of the included studies on the association between depression and PE.

**Figure 3:**
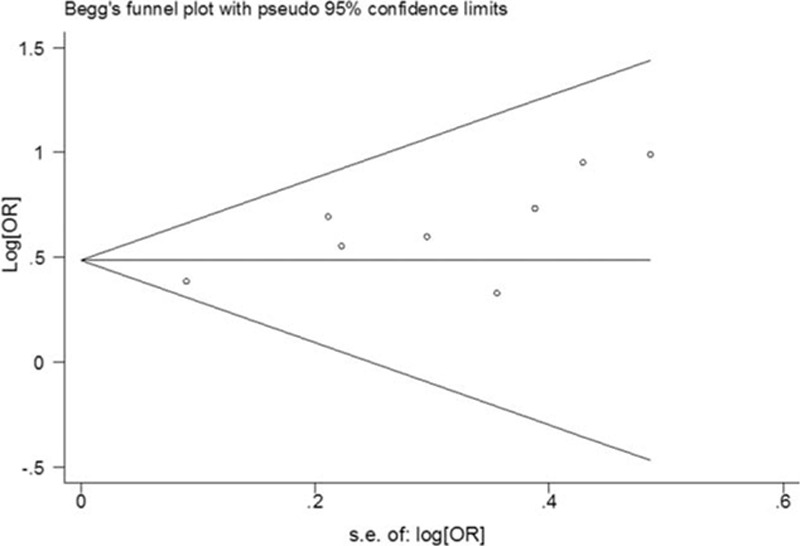
Funnel plot to detect publication bias.

Analysis of all 8 studies showed that depression was associated with a statistically significant increased risk of PE, compared with no depression (OR = 1.63, 95% CI:1.42–1.87). There was no evidence of between-study heterogeneity (*P* = 0.623, *I*^2^ = 0.0 %) (Fig. 2). In addition, we conducted subgroup meta-analysis by various study characteristics (Table 2). The pooled estimates of OR associated with PE in subgroups of studies according to mean age at baseline, geographical area, study design, sample size, controlling key confounders, and publication year. Depression significantly increased the risk of PE in all subgroups. Visual inspection of Begg's funnel plot did not identify substantial asymmetry (Fig. 3). There was no evidence of significant publication bias with Begg's test (*P* = 0.108).

**Table 2:**
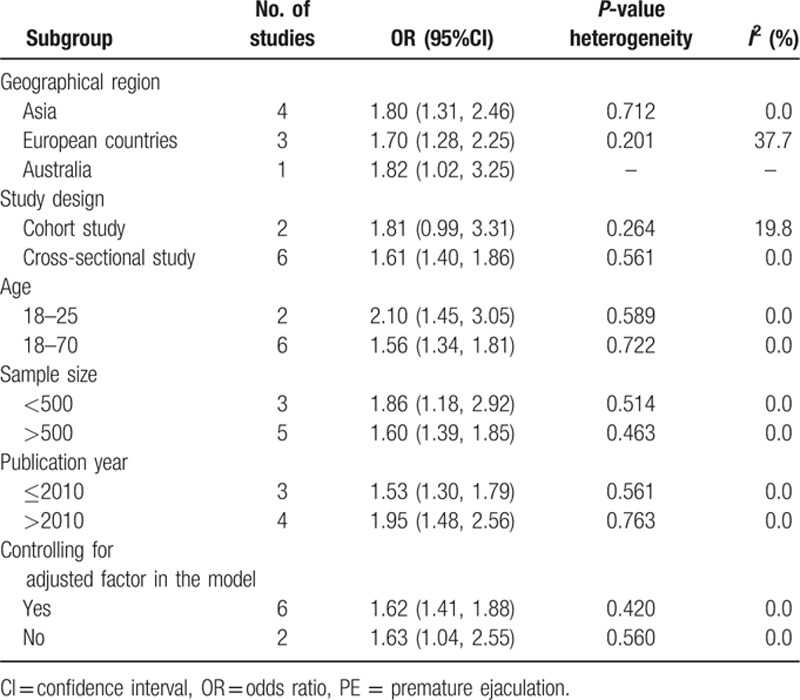
Subgroup analysis of the association between depression and PE.

## 4 Discussion

In our systematic review, we included 8 trials that met the quality criteria of patient selection. The present meta-analysis firstly evaluated available data on the relationship between depression and PE and showed that depression is associated with a significantly increased risk of PE. No relationship between age and the prevalence of PE among men >18 years of age was observed. Furthermore, the increased risk associated with PE persisted and remained statistically significant in all subgroup analyses stratified by various participant and study characteristics. Given that PE is prevalent worldwide, the findings of our meta-analysis have important implications for the evaluation and treatment of patients with PE.

PE is a multifactorial sexual dysfunction. Traditionally, the definition of PE mainly included objective physiological problems. Short IELT and loss of control were the most common syndromes. Gao et al^[23]^ reported that men with PE presented shorter IELT and lower International Index of Erectile Function 5 (IIEF-5) scores. In addition, higher rates of erectile dysfunction, anxiety, and depression were found in acquired PE patients. Modern evidence-based medicine has recognized that psychogenic causes have been suggested as important underlying conditions of PE in some cases. Thus, it is important to recognize that psychogenic effects could be secondary to PE as much evidence has documented a bidirectional relationship.

Previous studies have shown that negative psychological disorders might play an important role in precipitating or maintaining PE. In a multicenter and observational study conducted by Patrick et al^[24]^, more subjects in the PE group reported a greater level of distress than non-PE group (64% vs. 4%). Another cohort study of 334 Korean men explored the association between depression and PE.^[11]^ They found that self-assessed PE patients suffered from various psychological problems, such as depression, low self-esteem, bother, and low sexual satisfaction. It was likely that men with depression suffered higher levels of partner frustration, lack of confidence, or disappointment.

Depression is a common psychological disorder among patients with PE. The relationship between depression and PE might be bidirectional. There might exist some common pathophysiologic basis for the 2 disorders. Depression impairs male sexual function and couples’ sexual relationships. The poor sexual functioning and satisfaction can produce feelings of frustration and anxiety between partners. It may trigger low mood building up to the development of depressive disorder.^[25]^ However, some studies did not find a positive association between depression symptoms and PE.^[26]^ It has been observed that the neurobiological nature of PE possibly surpassed the importance of psychological factors.^[27]^ Furthermore, drug therapy, such as selective serotonin reuptake inhibitors and the tricyclic antidepressant clomipramine, were used to treat depression. The use of drug can delay ejaculation or improve PE.^[28]^

The underlying mechanism of how depression contributes to PE has not been fully elucidated. PE affects numerous aspects of a man's life, including sexual confidence, interpersonal relationships, and couples’ sexual relationships. Men with PE suffer a variety of negative effects including impaired quality of life, poor self-esteem, and lack of sexual confidence.^[8,29,30]^ Lack of ejaculatory control resulted in dissatisfaction with intercourse and increased emotional distress, and wide-ranging impact for both men with PE and their partners. PE has a significant psychological burden on men. A poor relationship may also be the case that leads to PE.

Chronic prostatitis symptoms may be an important organic cause of PE.^[26,31]^ Results from a population-based cross-sectional study by Mehik et al^[32]^ showed that from 26.2% to 42.5% of men with chronic prostatitis or chronic pelvic pain syndrome experienced PE. Other studies have suggested that PE is associated with chronic pelvic pain syndrome, prostatic infection, and prostatitis.^[33–35]^ Zhang et al^[26]^ showed that the chronic prostatitis symptom scores and IIEF-5 scores were the risk factors of depression in men with PE. From the current findings, prostatitis symptoms may negatively affected a patients’ mood, which might then induce a psychological burden and aggravate emotional and physical pain in PE patients.

The use of tobacco use and drugs were directly associated between depression and PE. The association between tobacco and sexual dysfunction has been well described as several unhealthy lifestyle factors.^[36]^ The persistent association existed among frequently used substances, such as tobacco or drugs, depression, and PE. We can assume that depression or PE men are also taking these most frequently used substances. Even if depression is not directly associated with PE, the link exists through tobacco and drug use.

Several limitations in our study should be recognized. First, the studies included in the analysis reached similar conclusions; however, they had dramatic variation in the methodologies, including different study designs and sample size. Second, sensitivity analysis and meta-regression analysis were not performed, because no significant difference was found between groups in the subgroup analyses. Third, we were unable to analyze the association between the severity of depression and PE, because these data were unavailable in most of the studies. Four, the definitions of depression and PE in the included studies were extremely variable, which may cause misclassification bias and heterogeneity.

In conclusion, this meta-analysis of observational studies suggests that depression is significantly associated with an increased risk of PE. Prevention and treatment of depression may substantially decrease the risk of PE. Regarding future research, additional experimental and high-quality prospective studies are required to clarify the potential mechanism linking depressive disorder and PE.

## Supplementary Material

Supplemental Digital Content
