# Supplementary material for: Relationship between premature ejaculation and depression: A PRISMA-compliant systematic review and meta-analysis
Source: Medicine (Baltimore). 2016 Sep 2;95(35):e4620. doi: 10.1097/MD.0000000000004620 (PMC5008563; doi:10.1097/MD.0000000000004620)
Supplement: Supplemental Digital Content [file medi-95-e4620-s001.doc]

| Box 1. Search strategy and terms used to identify studies on premature ejaculation and depression.  PubMed term search:  1. “premature ejaculation”  2. “sexual dysfunction”  3. “depression”  4. “depressive disorder”  5. “depress”  6. Search strategy for PubMed: (((premature ejaculation[Title/Abstract]) OR sexual dysfunction[Title/Abstract])) AND (((depressive disorder[Title/Abstract]) OR depress[Title/Abstract]) OR depression[Title/Abstract])  Embase term search:  1. “premature ejaculation”  2. “sexual dysfunction”  3. “depression”  4. “depressive disorder”  5. “depress”  6. Embase term search: ((1) OR 2) AND ((((3) OR 4) OR 5))  Cochrane Library search:  1. “premature ejaculation”  2. “sexual dysfunction”  3. “depression”  4. “depressive disorder”  5. “depress”  6. ((((depressive disorder in Title, Abstract, Keywords) OR depress in Title, Abstract, Keywords) OR depression in Title, Abstract, Keywords)) AND ((sexual dysfunction in Title, Abstract, Keywords) OR premature ejaculation in Title, Abstract, Keywords) |
| --- |
